# Effect of left atrial ligation-driven altered inflow hemodynamics on embryonic heart development: clues for prenatal progression of hypoplastic left heart syndrome

**DOI:** 10.1007/s10237-020-01413-5

**Published:** 2021-01-22

**Authors:** Huseyin Enes Salman, Maha Alser, Akshay Shekhar, Russell A. Gould, Fatiha M. Benslimane, Jonathan T. Butcher, Huseyin C. Yalcin

**Affiliations:** 1 Biomedical Research Center, Qatar University, Doha, Qatar; 2 Department of Mechanical Engineering, TOBB University of Economics and Technology, Ankara, Turkey; 3 Department of Biomedical Engineering, Cornell University, Ithaca, NY USA; 4 Regeneron Pharmaceuticals, Tarrytown, NY USA

**Keywords:** Left atrial ligation, Chick embryo, Hypoplastic left heart syndrome, Mitral valve, Left ventricle, Shear stress, Embryonic heart development, Computational fluid dynamics, Hemodynamics, Mechanobiology

## Abstract

Congenital heart defects (CHDs) are abnormalities in the heart structure present at birth. One important condition is hypoplastic left heart syndrome (HLHS) where severely underdeveloped left ventricle (LV) cannot support systemic circulation. HLHS usually initiates as localized tissue malformations with no underlying genetic cause, suggesting that disturbed hemodynamics contribute to the embryonic development of these defects. Left atrial ligation (LAL) is a surgical procedure on embryonic chick resulting in a phenotype resembling clinical HLHS. In this study, we investigated disturbed hemodynamics and deteriorated cardiac growth following LAL to investigate possible mechanobiological mechanisms for the embryonic development of HLHS. We integrated techniques such as echocardiography, micro-CT and computational fluid dynamics (CFD) for these analyses. Specifically, LAL procedure causes an immediate flow disturbance over atrioventricular (AV) cushions. At later stages after the heart septation, it causes hemodynamic disturbances in LV. As a consequence of the LAL procedure, the left-AV canal and LV volume decrease in size, and in the opposite way, the right-AV canal and right ventricle volume increase. According to our CFD analysis, LAL results in an immediate decrease in the left AV canal WSS levels for 3.5-day (HH21) pre-septated hearts. For 7-day post-septated hearts (HH30), LAL leads to further reduction in WSS levels in the left AV canal, and relatively increased WSS levels in the right AV canal. This study demonstrates the critical importance of the disturbed hemodynamics during the heart valve and ventricle development.

## Introduction

The mammalian heart is the first functional organ that develops during embryogenesis. During early development, diffusion is a sufficient means of transport for oxygen, nutrients, metabolic wastes, and hormones (Burggren 2004). To support organismal growth, it is well-understood that the heart pumps a continuous supply of blood and nutrients to extracardiac tissues. However, less is known about how intracardiac hemodynamic forces contribute to heart development and function, and how the dysregulation of such forces can contribute to the formation of congenital heart defects (CHDs).

The heart dynamically grows from a linear valve-less tube to a multi-chambered mature structure with 4 fibrous valves (Bartman and Hove 2005; Srivastava and Olson 2000). The heart continually pumps blood while growing and remodeling, which suggests that hemodynamic stresses within the heart may provide morphogenic cues to guide chamber development (Culver and Dickinson 2010; Forouhar et al. 2006; Hove et al. 2003; Yalcin et al. 2011). Several studies have cataloged the increasing hemodynamic burden on cardiac morphogenesis during which the heart grows over 100-fold in size (Butcher et al. 2007; Clark et al. 1989; Hu and Clark 1989). As a result of this dynamic hemodynamic environment, there are alterations in multiple mechanical signals (hydrostatic pressure, strain, fluid shear, etc.) in the heart. These changes accompany cardiac morphogenesis and regulate molecular and cellular responses that help coordinate downstream tissue remodeling.

CHDs form when cardiac morphogenetic processes are disrupted. Serious types of CHDs include tetralogy of fallot, hypoplastic left heart, transposition of great arteries, double outlet right ventricle, aortic stenosis, ventricular septal defect, and bicuspid aortic valve (Hoffman and Kaplan 2002). CHDs are seen in approximately at every 10–12 per 1000 live births throughout the world (Hoffman 2013). CHDs are the leading cause of death in infants under 1 year of age (Roger et al. 2011). Hypoplastic left heart syndrome (HLHS), in particular is a very severe condition and if left untreated, it is responsible for 25–40% of all neonatal cardiac deaths (Saraf et al. 2019). Despite their prevalence, the etiology of many CHDs remains unknown. There is indeed a genetic component. Zaidi and colleagues identified new point mutations in hundreds of genes that may contribute to 10% of CHDs (Zaidi et al. 2013). Strikingly, Øyen et al. (2009) reported that only 2–4% of infants with a CHD had a prior history of CHDs in their family, suggesting that CHDs mostly occur in patients without a family history of disease. This data suggests that, in addition to inherited factors, acquired factors also play a role in CHD formation. In fact, disturbed hemodynamics due to the mechanical perturbation of blood flow are shown to induce cardiac disease phenotypes (deAlmeida et al. 2007; Groenendijk et al. 2005; Hogers et al. 1999; Miller et al. 2003; Reckova et al. 2003; Sedmera et al. 1999, 2002). These studies suggest abnormal hemodynamic forces are among one of the non-genetic sources of CHDs.

Embryonic animal models have been widely used to study cardiac development and interrogate mechanisms that drive CHDs. Vertebrate species are preferred models since cardiac developmental processes are highly conserved. Typically studied models include mice (Li et al. 2015), zebrafish (Benslimane et al. 2020; Eisa-Beygi et al. 2018; Salman and Yalcin 2020; Zakaria et al. 2018), and chicken (or in general avian) embryos (Eisa-Beygi et al. 2018; Henning et al. 2011; Yalcin et al. 2010a, 2017). Chick embryo, in particular, is often used as a biological model of cardiac development due to several reasons including: the planar orientation of the embryogenesis on top of the yolk and easy embryo access for imaging and surgical modulation. The avian cardiogenic period is longer than other species (fish, frog, mouse) which enables more detailed spatiotemporal analysis and tolerance to microsurgical treatments. Moreover, chick embryos have fewer ethical concerns and closely resemble human cardiogenesis. The chicken embryo has therefore been extensively used to study the effects of hemodynamic alterations on cardiac development.

Microsurgical procedures for hemodynamic intervention in the chick embryo are designed to alter cardiac preload, afterload, or yolk sac vascular network. The primary surgical cardiac interventions in the chick embryo are vitelline vein ligation (VVL), outflow tract banding (OTB), and left atrial ligation (LAL). In LAL, a suture loop is placed around the left atrium and then is tied to constrict the left atrioventricular orifice and to decrease the effective volume of the left atrium (Sedmera et al. 1999). LAL has been performed at HH21–HH23 during the looping stages which is before ventricular septation. The partial ligation of the left atrium reduces its size, narrows the inflow area of the left ventricle (LV), and shunts blood flow from the left to the right side of the heart. The redistributed hemodynamic load results in underdevelopment in the left side and overdevelopment in the right side of the heart (Kowalski et al. 2014; Lindsey et al. 2014). The left side hypoplasia is a characteristic feature of HLHS. Therefore, the resulting phenotype due to LAL is accepted as the embryonic animal model of HLHS. The altered flow patterns induced by LAL interfere with normal looping, septation, and valvular formation to produce cardiac malformations. Chronic reduced preload and ventricular blood volume following LAL has been shown to modify the myocardial architecture prior to the development of cardiac defects. In particular, changes in the myofiber angle distribution, ventricular wall stiffness, compact myocardium thickness, and ventricular dimensions have been documented (Sedmera et al. 1999, 2002; Tobita et al. 2005; Tobita and Keller 2000; Tobita et al. 2002).

While there are several works in the literature investigating the disturbed hemodynamics following VVL and CTB (Espinosa et al. 2018; Hogers et al. 1997; Rugonyi et al. 2008; Shi et al. 2013), little has been reported for LAL. A comprehensive understanding of cardiac hemodynamics in the developing avian heart following LAL would enhance our current understanding of HLHS development. As shown by us and others, Doppler ultrasound or Doppler optical coherence tomography (Doppler OCT) can be used to measure cardiac flow velocities in embryonic chick for assessing heart function (Bharadwaj et al. 2012; Davis et al. 2009; Li et al. 2012; Oosterbaan et al. 2009; Yalcin et al. 2011). From these measurements, wall shear stress (WSS), which is an important regulator for heart development, and blood flow rates can be calculated with simplifying assumptions (Lindsey et al. 2015). For more accurate hemodynamic assessment, computational fluid dynamics (CFD) modeling is a useful tool for elucidating complex fluid motions, where the experimental measurement schemes would provide only limited information (Salman et al. 2019). We have previously developed a CFD approach to rigorously quantify the evolving hemodynamic environment of the atrioventricular (AV) and outflow tract (OFT) canals of avian embryos (Bharadwaj et al. 2012; Yalcin et al. 2011). We used model geometries generated using micro-CT images and ultrasound measured blood flow velocities inputted as inlet velocities (Benslimane et al. 2019). Such computational techniques have been applied to investigate disturbed hemodynamics following VVL (Groenendijk et al. 2005), and OTB (Liu et al. 2007; Menon et al. 2015). Recently, blood flow alteration following LAL were examined as well using a virtual in silico approach (Kowalski et al. 2014). These computational studies reported that heart development is sensitive to altered biomechanical stimulus, and abnormal WSS impact the initiation and progression of the cardiac defects.

To model the comprehensive hemodynamic patterning during HLHS development, we made use of the LAL chick model and use CFD modeling to unravel blood flow alterations in the AV canal (i.e., mitral and tricuspid valve forming region). The hemodynamic analyses are performed immediately after LAL (HH25) and after the completion of AV canal septation into left and right (HH30). We quantified morphological abnormalities in LAL embryos from micro-CT geometries to correlate these with disturbed hemodynamic forces. According to the findings, LAL interference significantly altered the hemodynamic environment in the heart and initiated a WSS unbalance between the left and right sides of AV cushions. The disruption in the left side of the heart cannot be recovered during the embryonic development. This information is critical to understand CHD development and hence for the generation of future clinical therapies for CHDs.

## Materials and methods

### Embryo culture, transport, and environmental stabilization

**Fig. 1:**
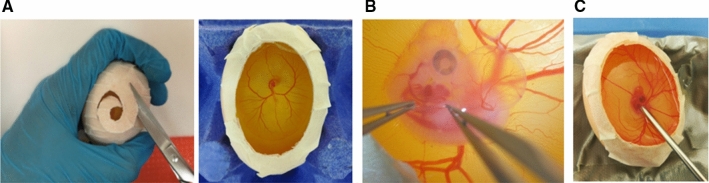
In-ovo chick embryo culture enables access to surgeries and analysis. **a** Embryos are cultures within their shells and opened at embryonic day (ED) 3. The egg opening creates an easy access for **b** surgery and **c** echocardiographic imaging, respectively

Embryonic chicks grow for up to 5 days on top of the yolk sac before sinking into the middle of the egg. These embryos rest on their left side and grow in planar fashion. Fertilized eggs are cultured as shown in our previous methodology publication (Benslimane et al. 2019). In short, eggs are cultured at 37.5 °C, 60% humidity, and continuous rocking for 72 h. On the 2.5th–3rd day, the eggs are opened, and the hole is covered. The eggs are kept in a portable incubator and kept until day 3.5 to pursue with the LAL surgery. This in-ovo culture technique enables full access to the embryos for surgeries (Fig. 1b). Finally, by using the surface tension of a small amount of warmed sterile Tyrode’s solution, we are able to develop a fully hydrated zone between the imaging device and the embryo, thus removing the need for toxic ultrasound gels or driving the microscope objective into the embryo (Fig. 1c). The end result is the ability to quantitatively image the embryos over a large period of development (HH12–HH36+).

### LAL procedure

**Fig. 2:**
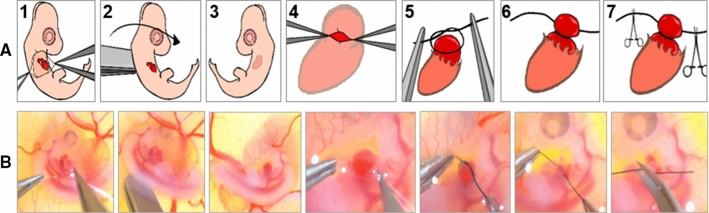
LAL surgery. **a** Schematic representation of LAL steps. **b** Representative pictures of LAL steps. *Step 1* Opening chorionic and allantoic membranes and exposing the animal. *Step 2* Flipping the animal vertically to expose its left side shown in *Step 3*. *Step 4* Opening the pericardium and exposing the left atrium. *Step 5* Placing the pre-prepared surgical knot on the top of the atrium. *Step 6* Tightening the knot around the atrium. *Step 7* Cutting the extra ends of the suture and finally flipping back the animal to its original orientation

LAL procedure is performed at 3.5th day (HH21) of incubation (Fig. 2). In this technique, the first step is locally removing chorionic and allantoic membranes over the embryo grown in-ovo. The embryo is then lifted and rotated vertically so that the left side is now exposed. After that, using fine forceps, the pericardium over the left atrium is opened. Previously prepared ~ 0.5 mm diameter knots from 10-0 nylon surgical sutures are placed over the left atrium. These knots are then tightened so that the left atrium volume is reduced by about 75%. This interference is expected to constrict the blood flow through the left side of the AV canal. The edges of the knot are cut with micro-scissors and excess suture is carefully removed. Finally, the embryo is flipped back to its original position where the right side is on top. Details of this procedure are visualized in Fig. 2 and can be seen in our video protocol (Yalcin et al. 2010b).

### In vivo hemodynamic measurements via echocardiography

**Fig. 3:**
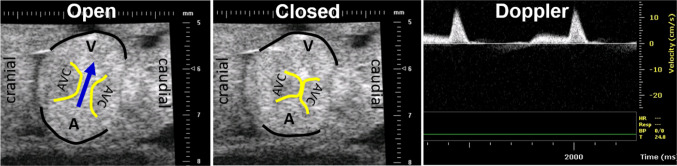
Echocardiography imaging for an HH21 embryo. B-mode images and Doppler velocity measurements for AV canal. In B-mode images, AVC is atrioventricular cushion. Arrow shows the blood flow direction. Edges of atrial and ventricular myocardium are highlighted in black. Edges of AVC are highlighted in yellow. Doppler blood velocity profile shows two peaks which correspond to initial ventricular relaxation and atrial contraction/ventricular filling phases

Ultrasound imaging is performed to monitor hemodynamic changes following the LAL interference as previously described (Butcher et al. 2007; McQuinn et al. 2007). Imaging is performed using a 55 MHz RMV704 and 30 MHz RMV707 scanhead on the Vevo770 high-frequency ultrasound system (VisualSonics, Toronto, Canada). The temperature of embryos during ultrasonography is closely monitored and kept constant at 37.5 °C using a water circulating heater. For imaging, an aqueous contact zone is made between the ultrasound probe and the embryo using warmed Tyrode-HEPES solution. B-mode and Doppler velocity profiles of the AV canal, and proximal outflow are acquired according to our previously published techniques (Fig. 3) (Benslimane et al. 2019; Butcher et al. 2007). Cardiac output, stroke volume and ejection fraction are quantified using the method of DeGroff (DeGroff 2002). Ultrasound measurements are performed on the 3.5-day (HH21) embryos that are just subjected to interference (5–6 h after the LAL procedure) and on the 7-day (HH30) embryos, in which the AV canal septated into left and right canals. For each stage, 6 embryos are analyzed.

### Histology

Embryonic hearts are isolated 24-h post-LAL (HH25) and 72-h post-LAL (HH30), and fixed along with controls by incubation in 4% paraformaldehyde (PFA) for 24 h at 4 °C with continuous rotation. The hearts are serially dehydrated in ethanol (70–100%, 1 h per concentration), cleared twice with xylene, and paraffin processed. The hearts are cut into 10 µm thick sagittal sections. The sections are stained following the common H&E procedure, mounted by DPX mounting media, and covered with a cover slip. The slides are then left on a slide warmer overnight. The sections are examined under stemi-508 stereomicroscope; pictures are taken at 2.5X and 2X magnification, respectively. Pictures of 3 independent hearts of each treatment are taken and different parameters are quantified using ImageJ.

### Microfil cast creation and micro-CT imaging

**Fig. 4:**
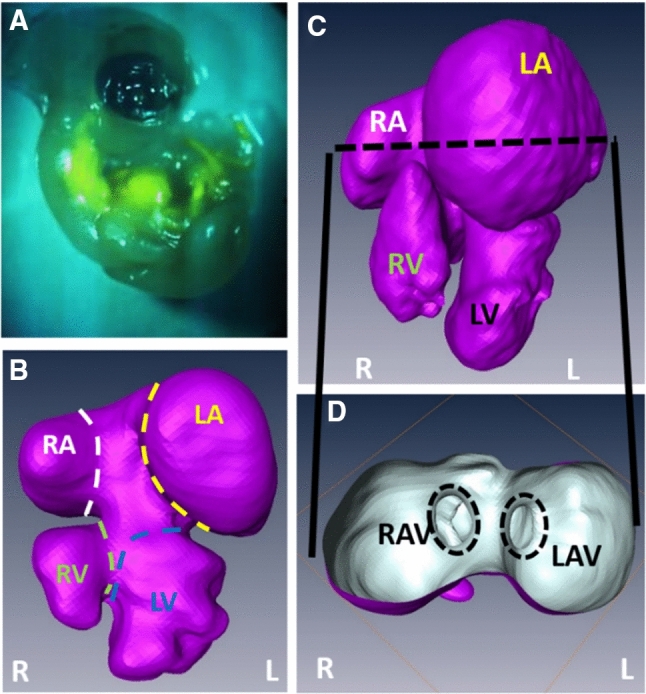
Micro-CT cast creation and 3D volume generation. **a** CT-dense contrast agent is perfused through cardiovascular system for cast creation. Micro-CT scanning enables generation of 3D heart volumes. **b** Unseptated 3.5-day (HH21) heart. **c** Septated 7-day (HH30) heart. **d** Orifice sizes for right and left AV canals in **c** can be seen through a section inside atria. *RAV* right atrioventricular canal, *LAV* left atrioventricular canal, *R* right, *L* left, *RA* right atria, *LA* left atria, *RV* right ventricle, *LV* left ventricle

Microfilm casts are created for embryonic hearts by perfusing Microfil (Flow-Tech, Carver, MA) into microvascular lumens through drawn glass capillary micro-needles (Butcher et al. 2007). In this technique, Microfil solution polymerizes into a cast, preserving the cardiac chambers at physiologically dilated volume with open valves. Microfil perfused embryonic bodies are dissected away from the vitelline network and placed in 3 ml tubes filled 4% PFA and preserved in 4 °C until scanning (Fig. 4a). The embryos are then scanned via micro-CT at 10 µm voxel resolution (approximately 400 slices/embryo) using GE micro-CT scanner (GE Healthcare eXplore CT 120). An initial scout image is generated at low resolution to confine the high-resolution scan to adjust the heart region. The datasets are reconstructed into 3D geometry using Mimics (Materialise, Leuven, Belgium) software. The cardiac anatomy is segmented as previously described (Butcher et al. 2007). These 3D geometries are used to quantify ventricular chamber (Fig. 4b and c) and AV valve orifice sizes (Fig. 4d) for LAL and control embryos. These reconstructed geometries are also used in the CFD models. Micro-CT scanning is performed on the embryos that are just subjected to interference (5–6 h after the LAL procedure) and on 7-day (HH30) embryos, in which the AV canal septated into left and right canals. For each stage, 6 embryos are filled and scanned for further analysis.

### CFD model generation and simulations

**Fig. 5:**
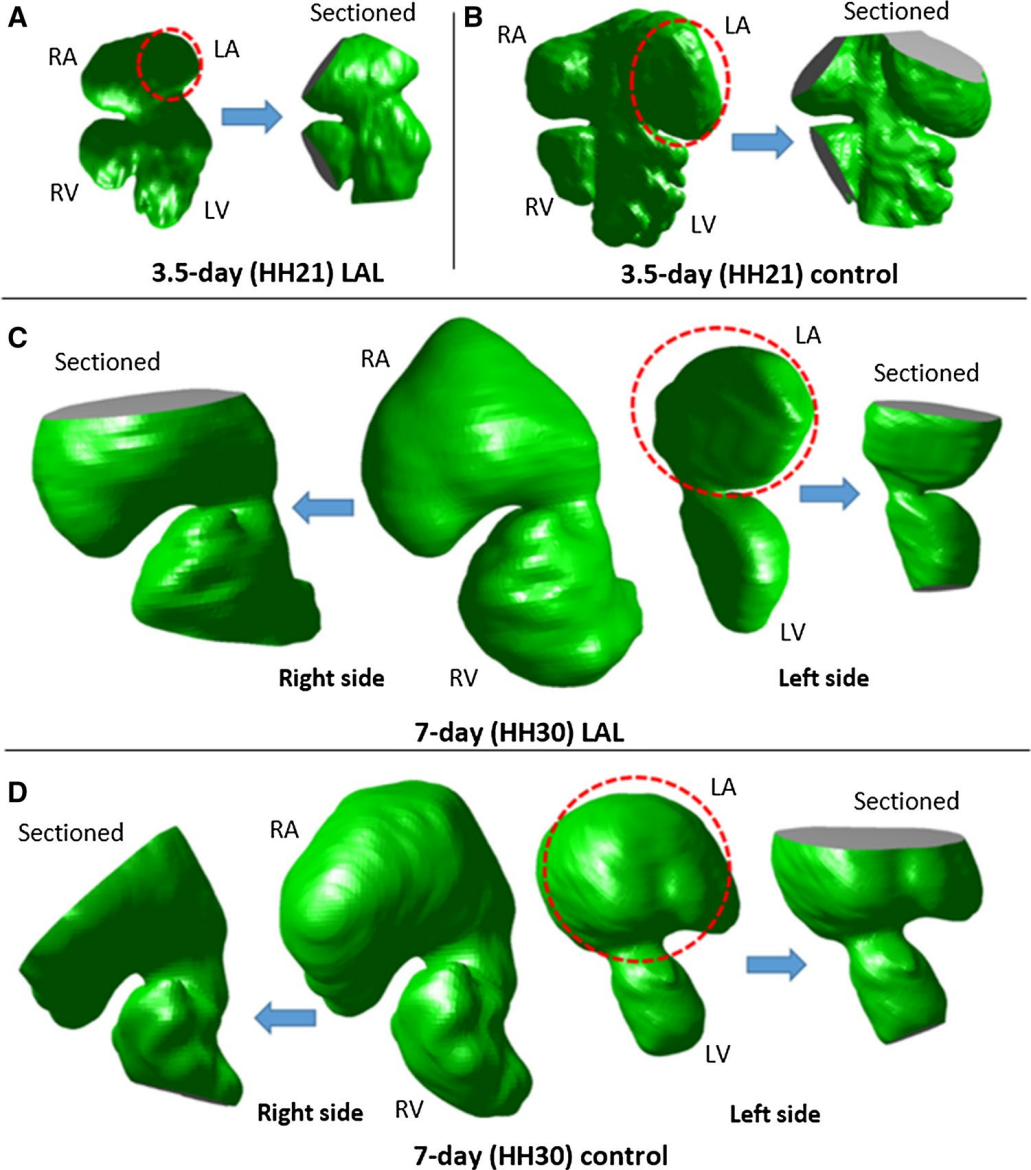
3D geometries and sectioned models. **a** HH21 LAL hearts. **b** HH21 control hearts. **c** HH30 LAL hearts. **d** HH30 control hearts. Left atria is outlined by dash line for all embryonic hearts. LA, LV, RA, RV are sectioned to apply the inlet and outlet boundary conditions. *LA* left atrium, *RA* right atrium, *LV* left ventricle, *RV* right ventricle

STL surface files are first converted to IGES files and then imported into ANSYS Workbench 19.2 (Canonsburg, PA, USA) for meshing. The atrium, ventricle, and AV canal lumen are isolated for 3.5-day (HH21) and 7-day (HH30) hearts (Fig. 5). For HH30 embryos, left AV canal and right AV canal are separated (Fig. 5c and d). The atriums are virtually sectioned about 50% through their height to simulate the inflow conditions. The apex regions of the ventricles are also sectioned for simulating the blood filling. For HH21 embryos, both left and right inlets and outlets are created on the same geometry. For HH30 embryos, left AV canal and right AV canal are modeled separately, due to the septation of the AV canal. For each stage, 3 embryos are analyzed.

These static geometries are generated to approximate the instant of systolic inflow where peak flow occurs at AV canal. The geometries include 3D constructed anatomical features of the AV canal. The geometries are meshed using ANSYS Workbench and meshed anatomies are then imported into ANSYS Fluent, which is a CFD software capable of solving transient 3D flow dynamics. In the simulations, we use the stage specific pulsatile flow velocity profiles which are determined from the Doppler ultrasound measurements, and non-Newtonian blood rheology from our previous studies (Yalcin et al. 2011). We approximate the inflow and outflow zones as flat elliptical regions on the mid-atrial and distal ventricle cross sections, respectively. For simulating the initial blood flow profiles from the mid-atria, we employ an iterative boundary approach as previously described (Yalcin et al. 2011) using Doppler ultrasound quantified middle-AV cushion velocity profiles as the in vivo baseline. Briefly, CFD simulations are conducted using a magnitude-scaled middle-cushion flow profile as a “fictitious” middle-atrial input flow profile. The magnitude is iterated across a range of inlet velocities and CFD simulations are performed to develop a correlation between the atrial inflow and ultrasound measured middle-AV cushion velocity profiles. From this, we establish an input flow profile that generated the same middle-cushion outflow velocity profile as measured in vivo. These velocity profiles are then used as the atrial velocity input for the CFD simulations.

A mesh convergence study is performed, and it is found that at most 350,000 elements are sufficient for mesh independency of solutions for all processed stages. Therefore, all simulations are performed using approximately 350,000 tetrahedral elements. The simulations are performed during one complete cardiac cycle. The solution convergence is enforced by reducing the residual of the continuity equation, as well as x, y, and z-momentum to 10^−6^ for all time steps. Each CFD analysis is performed by employing 40 time steps with 0.015 s increments.

### Statistical analysis

All statistical analyses (for Doppler analysis and histology quantification) are conducted with two tail Student’s *t* test, and a *p* value which is less than 0.05 is considered statistically significant.

## Results

### LAL significantly alters AV canal hemodynamics at HH21 stage

**Fig. 6:**
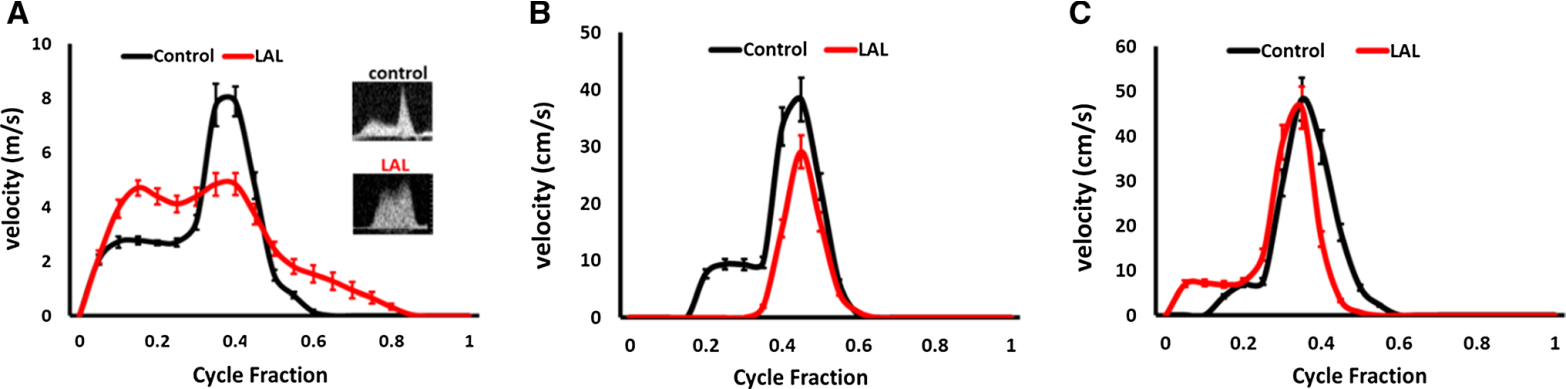
Hemodynamic profiles of LAL and control chicks. **a** HH21 AV canal velocity profiles. **b** HH30 L-AV canal velocity profiles. **c** HH30 R-AV canal velocity profiles. Results are presented as mean ± standard error of the mean (SEM). *L-AV* left atrioventricular canal, *R-AV* right atrioventricular canal

Chick embryos are cultured and undergone LAL surgery at HH21, and Doppler analysis is conducted to assess the hemodynamic alterations. Figure 6a shows the velocities at AV canal over cardiac cycle for control and LAL hearts. HH21 AV control velocity profile shows distinct peaks representing initial filling and atrial contractions, whereas AV LAL velocity profile is more spread with higher initial filling peak and lower atrial contraction peak. LAL results in an immediate decrease in peak velocity in AV canal from 8.1 ± 0.9 cm/s for controls to 4.84 ± 0.8 cm/s for LALs. Time averaged velocities (TAVs), on the other hand, are not different at AV canal, 2.27 ± 0.2 cm/s for controls and 2.05 ± 0.2 cm/s for LALs. Cardiac outputs are not different for control and LAL embryos (70 ± 1.5 µlt/min for controls, 76 ± 1.4 µlt/min for LALs). Stroke volume (0.67 ± 0.13 mm^3^ for controls, 0.7 ± 0.12 mm^3^ for LALs) and ejection fraction (63 ± 6% for controls, 61 ± 4% for LALs) are not different as well for control and LAL embryos. These results show that even though LAL causes an immediate decrease in peak velocity, it does not alter cardiac work (see Table 1 for the tabulated values of HH21 heart).

**Table 1:** HH21 AV canal velocities and heart function parameters

	Peak velocity at AV canal (cm/s)	TAV at AV canal (cm/s)	Cardiac output (µlt/min)	Stroke volume (mm^3^)	Ejection fraction (%)
Control	8.1 (0.9)	2.27 (0.2)	70 (1.5)	0.67 (0.13)	63 (6)
LAL	4.84 (0.8) ↓	2.05 (0.2)	76 (1.4)	0.70 (0.12)	61 (4)

The arrow indicates significant decrease in velocities. Standard error of the mean (SEM) is given in the parentheses

### LAL results in sustained hemodynamic alteration in the left AV canal but not in right AV canal at HH30

LAL surgery is performed on embryonic chicks at HH21 and these embryos are kept in culture until the Doppler analysis at HH30. Figure 6b shows the velocities at left atrioventricular (L-AV) canal over cardiac cycle for control and LAL hearts. Peak velocity in L-AV canal is determined as 38.7 ± 2.0 cm/s for control embryos at HH30. Peak velocity in L-AV canal decreases to 30.4 ± 3.0 cm/s for HH30 LAL embryos. TAV in L-AV canal is obtained as 5.4 ± 0.8 cm/s for controls and 3.4 ± 0.9 cm/s for LALs (see Table 2 for tabulated values of HH30 L-AV). Figure 6c shows the velocities at right atrioventricular (R-AV) canal over cardiac cycle for control and LAL hearts. At HH30, peak velocity in R-AV canal is not different for control and LAL embryos (48.9 ± 2.4 cm/s for controls, 45.4 ± 3.0 cm/s for LALs). TAV in R-AV canal is also not different for control and LAL embryos (6.3 ± 1.1 cm/s for controls, 6.5 ± 0.9 cm/s for LALs). There is a clear decrease in velocity levels in L-AV canal and no significant change in R-AV canal.

**Table 2:** HH30 AV canal velocities and heart function parameters

	Peak velocity at AV canal (cm/s)	TAV at AV canal (cm/s)
Control—Left AV	38.7 (2.0)	5.4 (0.8)
LAL—Left AV	30.4 (3.0) ↓	3.4 (0.9) ↓
Control—Right AV	48.9 (2.4)	6.3 (1.1)
LAL—Right AV	45.4 (3.0)	6.5 (0.9)

Arrows indicate significant decrease in parameters. Standard error of the mean (SEM) is given in parentheses

### Histological analysis

**Fig. 7:**
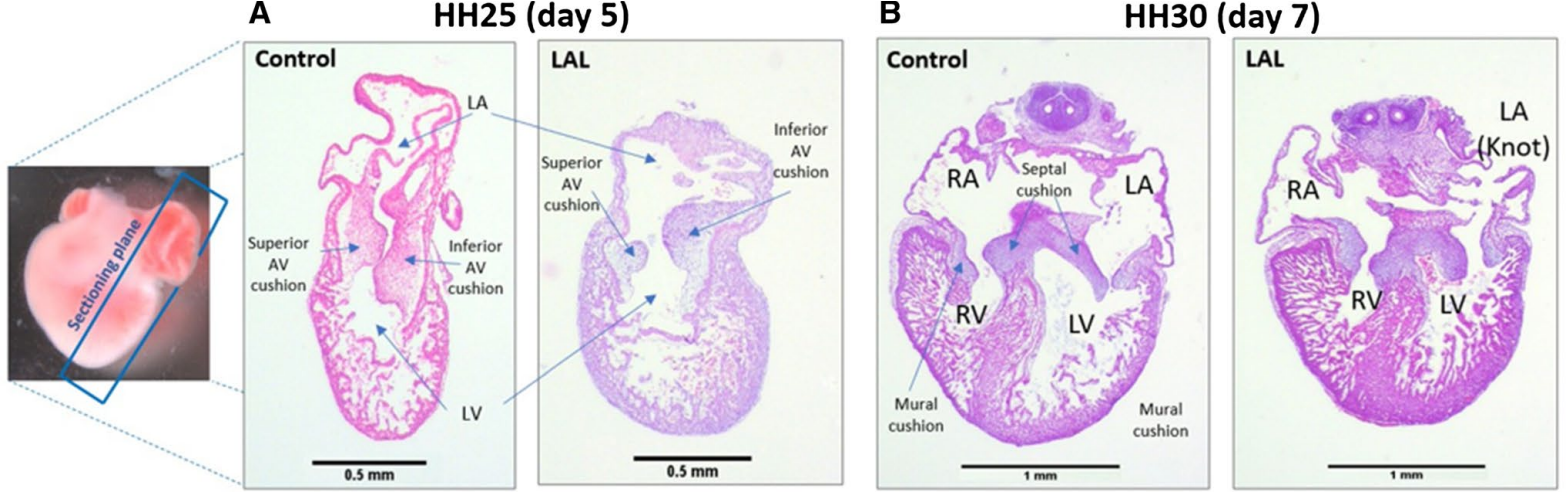
Example of histologically H&E stained slides of embryonic chick hearts at different stages. **a** The orientation and anatomy of HH25 for control and LAL hearts. **b** The anatomy of HH30 for control and LAL hearts

In order to examine the effect of altering hemodynamics on heart conformation and development, and to confirm the generation of HLHS, histological analysis is performed. Histological slides are prepared 24-h (HH25) and 72-h post-LAL (HH30) to validate the impact of LAL interference. Figure 7 shows an example of the histological slides from HH25 and HH30, and the anatomy of each stage.

**Fig. 8:**
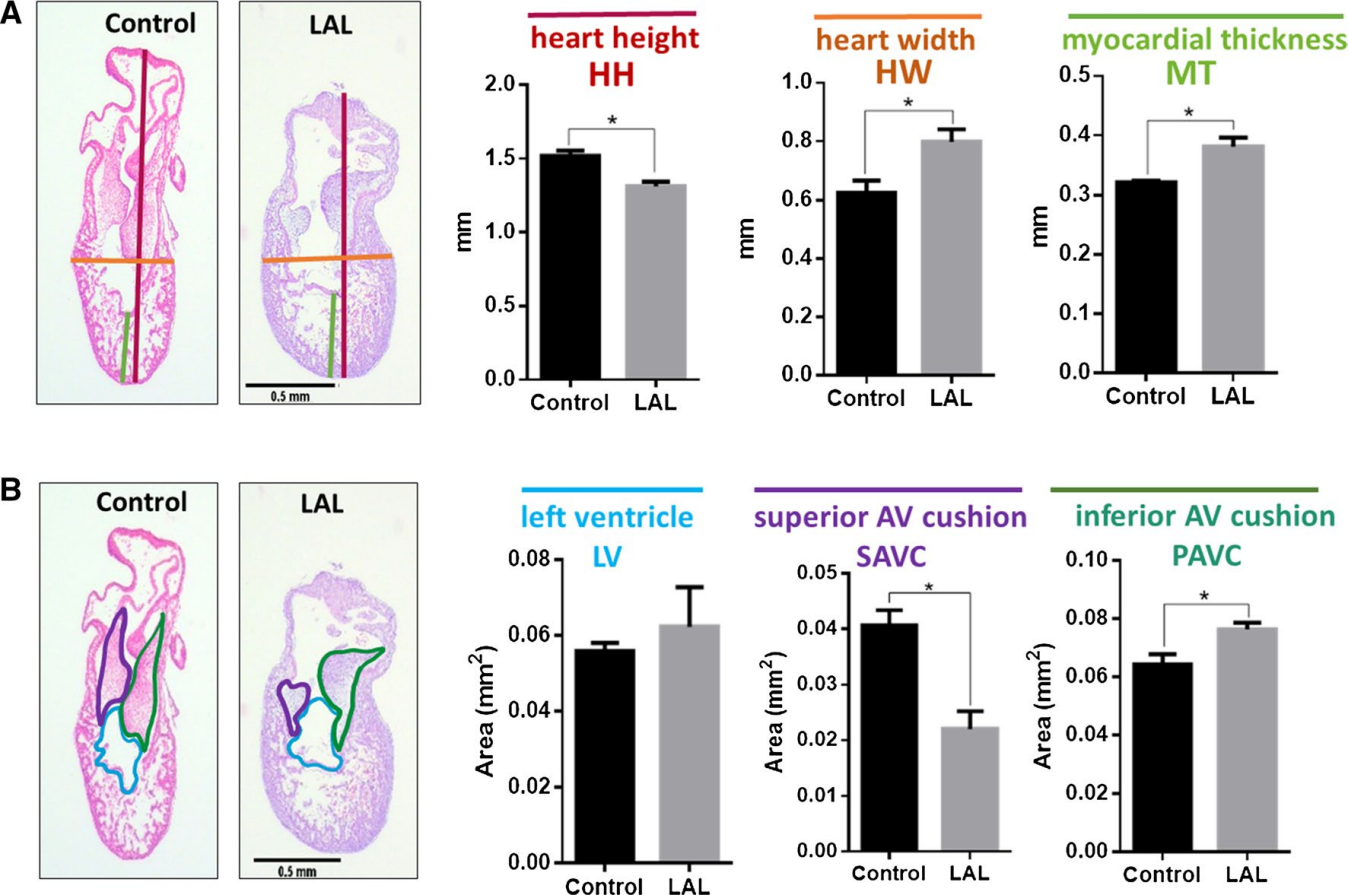
LAL causes anatomical changes on embryonic day-5 (ED5) chick hearts 24-h post-surgery. ED5 control and LAL hearts are oriented and H&E processed. **a** Quantification of length parameters (HH, HW, and MT) in both control and LAL hearts. **b** The quantification of area parameters (LV, SAVC, and IAVC) in both control and LAL hearts. *HH* heart height, *HW* heart width, *MT* myocardium thickness, *LV* left ventricle, *SAVC* superior atrioventricular cushion, *IAVC* inferior atrioventricular cushion. Data is presented as mean (*n* = 3), error bars indicate SEM, and *p *< 0.05 indicates statistical significance as determined by Student’s *t* test (*p < 0.05)

At HH25, the heart is still not septated, and the valves are still primitive cushions. The results suggest that LAL causes immediate change in the heart size as presented in Fig. 8. The hearts become shorter (*p* = 0.012) and wider (*p* = 0.044) as a consequence of LAL. Since the assessment is done only 24-h post-LAL, LV size does not show significant reduction (see Fig. 8b). However, superior AV cushion size is significantly reduced (*p* = 0.012). This suggests that the change in flow significantly affects the valve development at the embryonic level.

**Fig. 9:**
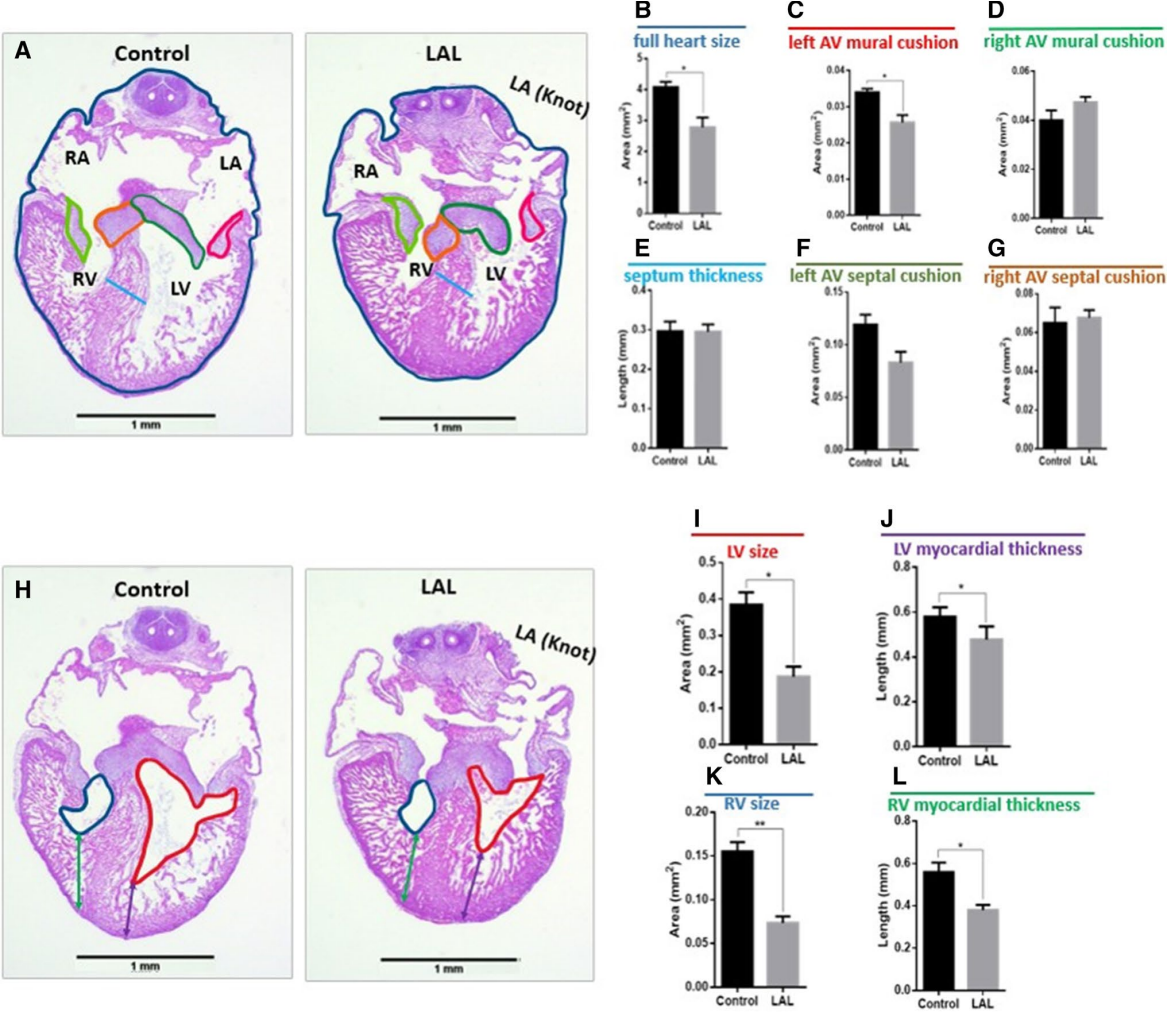
LAL causes anatomical changes on ED7 chick hearts 72-h post-surgery. ED7 control and LAL hearts are oriented and H&E processed. Different parameters are measured using ImageJ. **a** Demonstration of the quantified parameters shown in **b**–**g**. **h** Demonstration of the parameters quantified in **i**–**l**. *LA* left atrium, *LV* left ventricle, *RA* right atrium, *RV* right ventricle. Data is presented as mean (*n* = 3), error bars indicate SEM, and *p* < 0.05 indicates statistical significance as determined by Student’s *t* test (*p < 0.05)

Histological analysis is performed on hearts 72-h post-LAL (HH30). The result suggests that the full heart size is significantly reduced (*p* = 0.034) as shown in Fig. 9b. The valve leaflets are not significantly changed, but the L-AV mural valve is reduced significantly in the LAL hearts (see Fig. 9c). This confirms the severe effect of LAL on the valve development and confirms what we see in the Doppler analysis (see Fig. 6) and in the embryonic day-5 histology (see Fig. 8). Since the analysis is done 72-h post-surgery, HLHS is shown to be successfully generated as the left ventricle size shows severe reduction in LAL when compared to the control (see Fig. 9). In addition, the results indicate that the myocardial thickness significantly reduces with LAL. Surprisingly, RV thickness and right myocardial thickness also reduce, suggesting the altered hemodynamic parameters affect the whole heart development and change the cardiac output at the embryonic stage.

### AV valve orifice measurements via micro-CT

**Fig. 10:**
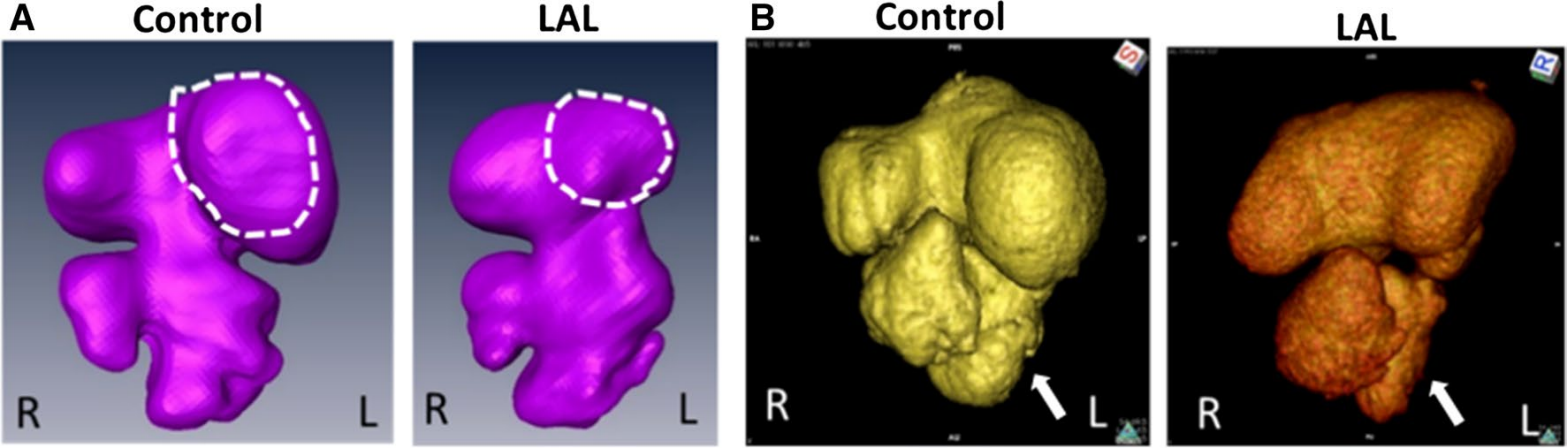
Micro-CT geometries for control and LAL embryos at **a** HH21 and at **b** HH30. Left atrium regions are outlined for HH21 embryos. Left ventricle regions are shown by arrows for HH30 embryos

In Fig. 10, the volumetric changes due to LAL are shown for HH21 and HH30 embryos. LV volume decreases from 2.26 ± 0.15 mm^3^ for controls to 1.69 ± 0.33 mm^3^ for LALs (25% decrease). RV volume increases from 2.37 ± 0.31 mm^3^ for controls to 2.81 ± 0.42 mm^3^ for LALs (19% increase). LA volume does not change and it is measured as 4.06 ± 0.52 mm^3^ for controls and 4.08 ± 0.75 mm^3^ for LALs. RA volume significantly increases from 5.3 ± 0.8 mm^3^ for controls to 7.7 ± 0.99 mm^3^ for LALs (45% increase). The left AV canal orifice area decreases from 0.34 ± 0.08 mm^2^ for controls to 0.28 ± 0.05 mm^2^ for LALs (18% decrease). The right AV canal orifice area increases from 0.35 ± 0.06 mm^2^ for controls to 0.42 ± 0.06 mm^2^ for LALs (20% increase). Left AV canal cardiac output dramatically decreases from 1.14 ± 0.21 ml/min for controls to 0.60 ± 0.13 ml/min for LALs (47% reduction). On the other hand, right AV canal cardiac output significantly increases from 1.37 ± 0.24 ml/min for controls to 1.67 ± 0.23 ml/min for LALs (22% increase). These results demonstrate that the flow is redistributed toward the right side after LAL, which is causing an enlargement in right AV valve orifice and a decrease in left AV valve orifice. All results are summarized in Table 3.

**Table 3:** Ventricular volumes, atrial volumes, and AV canal orifice sizes for control and LAL embryos at HH30

	LV volume (mm^3^)	RV volume (mm^3^)	LA volume (mm^3^)	RA volume (mm^3^)	Left AV canal orifice area (mm^2^)	Right AV canal orifice area (mm^2^)	Left AV canal cardiac output (ml/min)	Right AV canal cardiac output (ml/min)
Control	2.26 (0.15)	2.37 (0.31)	4.06 (0.52)	5.3 (0.8)	0.34 (0.08)	0.35 (0.06)	1.14 (0.21)	1.37 (0.24)
LAL	1.69 (0.33) ↓	2.81 (0.42) ↑	4.08 (0.75)	7.7 (0.99) ↑	0.28 (0.05)↓	0.42 (0.06) ↑	0.60 (0.13) ↓	1.67 (0.23) ↑

*LV* left ventricle, *RV* right ventricle, *LA* left atrium, *RA* right atrium

Arrows indicate significant change in parameters. Standard errors of the mean (SEM) are given in parentheses

### HH21 AV canal simulations

**Fig. 11:**
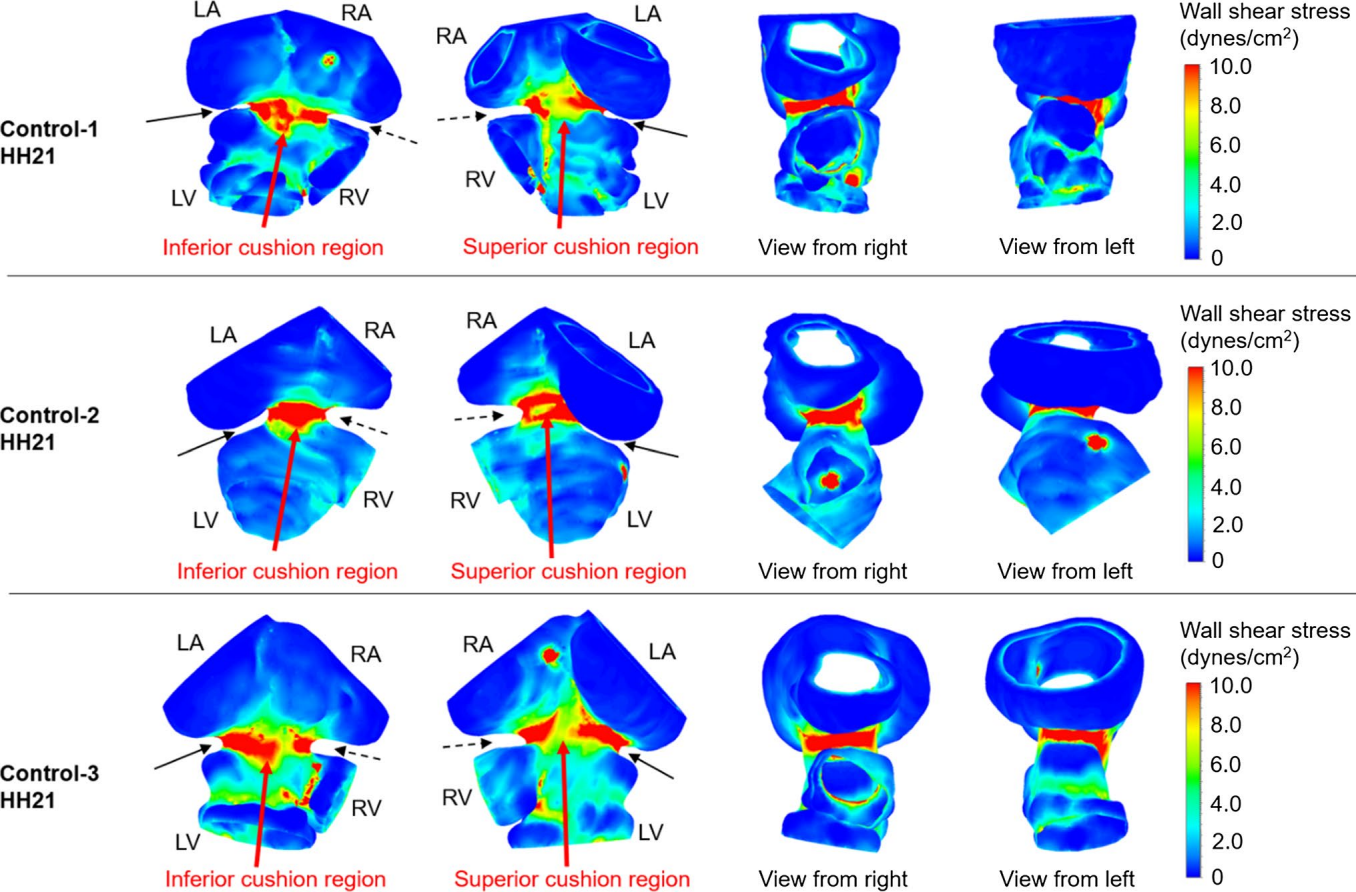
WSS distribution at peak AV flow velocity for HH21 control hearts. Solid and dashed arrows show left and right AV canal regions, respectively. Red arrows designate inferior and superior AV cushions

**Fig. 12:**
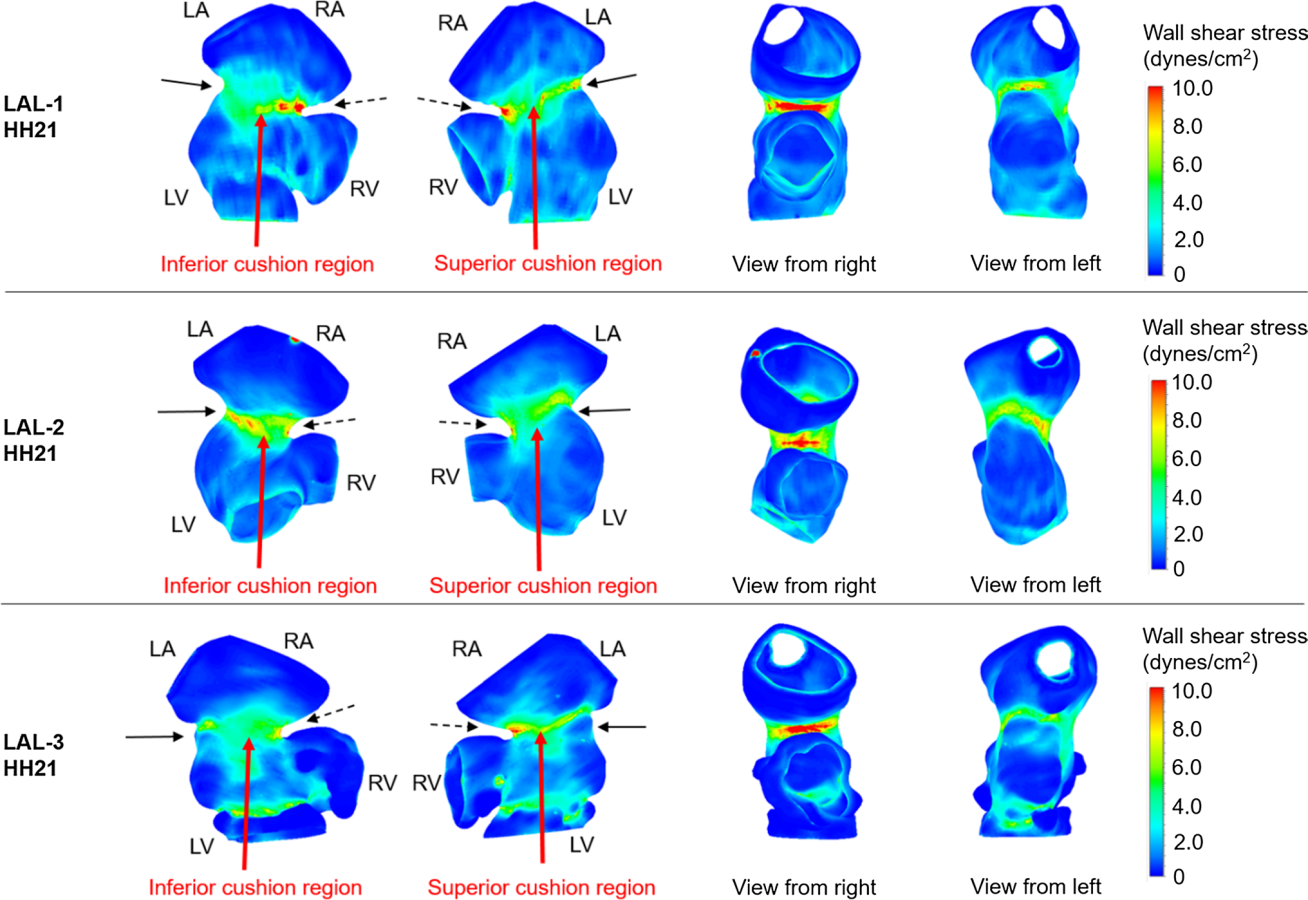
WSS distribution at peak AV flow velocity for HH21 LAL hearts. Solid and dashed arrows show left and right AV canal regions, respectively. Red arrows designate inferior and superior AV cushions

CFD models are generated for control and LAL embryos at HH21. The simulations are performed to investigate the immediate alterations in hemodynamics due to the LAL interference. Micro-CT scans are used for the generation of model geometries. Three LAL and three control geometries are generated for the simulations. Numerical results are determined by averaging the findings of three embryonic models. WSS distributions are investigated at the left and right side of the AV canal valvular regions as shown in Figs. 11 and 12 for control and LAL hearts, respectively. Inferior and superior cushion regions, consistent with AV cushion regions shown in Fig. 7, are designated with red arrows in Figs. 11 and 12. Left AV valve region eventually becomes the mitral valve and the right region becomes tricuspid valve as the heart develops. Peak WSS values on these regions are temporally averaged using peak values during one cardiac cycle. In further sections, we call these averaged peak values as peak WSS. Area-weighted average WSS values on these regions are determined for all time points and temporally averaged during one cardiac cycle. We call these area-weighted average values as average WSS.

According to HH21 simulations, LAL results in an immediate decrease in peak WSS in left AV canal from 1.16 ± 0.11 Pa for controls to 0.63 ± 0.076 Pa for LALs (45.7% decrease). Similarly, average WSS in left AV canal decreases from 0.14 ± 0.0075 Pa for controls to 0.115 ± 0.0056 Pa for LALs (17.9% decrease). In right AV canal, peak WSS for controls is 1.28 ± 0.2 Pa and peak WSS for LALs is 1.07 ± 0.078 Pa (16.4% decrease). Average WSS in right AV canal is 0.2 ± 0.018 Pa for controls and 0.188 ± 0.0056 Pa for LALs, where the difference is not significant. These results show that LAL results in an immediate decrease in WSS levels in the left side of the AV canal, while WSS in the right side is relatively unchanged compared to controls.

Completed histological assessments provide a deeper insight about the development of the superior and inferior AV cushions. Computationally analyzed WSS levels on the superior and inferior sides of the AV cushions of HH21 embryos confirm and validate the results of histological findings. Three embryos are used to determine the mean WSS on the superior and inferior sides of the AV cushions. The results are summarized in Table 4. WSS difference between the superior and inferior sides of LAL embryos is not significant (*p* = 0.518). For control embryos, this WSS difference between the superior and inferior sides is higher compared to LALs, but it is still not significant (*p* = 0.180).

**Table 4:** Mean WSS on the superior and inferior sides of HH21 embryos

	Control		LAL	
	Superior	Inferior	Superior	Inferior
Embryo 1	0.8	1.05	0.45	0.5
Embryo 2	0.8	1.7	0.5	0.6
Embryo 3	0.75	0.85	0.5	0.45
Mean	0.78	1.2	0.48	0.52
Standard deviation	0.029	0.44	0.029	0.076

WSS values are given in Pa

WSS change on inferior side between the control and LAL embryos is also found as not significant (*p* = 0.058). On the other hand, the WSS change on the superior side between the control and LAL embryos is determined as quite significant (*p* = 0.00022). This indicates that superior WSS levels are critically reduced for the LAL embryos. This finding supports the results of histological analysis, where the superior cushion size is found as significantly reduced for the LAL embryos. It is clearly understood that the reduced size of the superior AV cushions of LAL embryos has a direct relation with the significantly lowered WSS levels on the superior AV cushions.

**Fig. 13:**
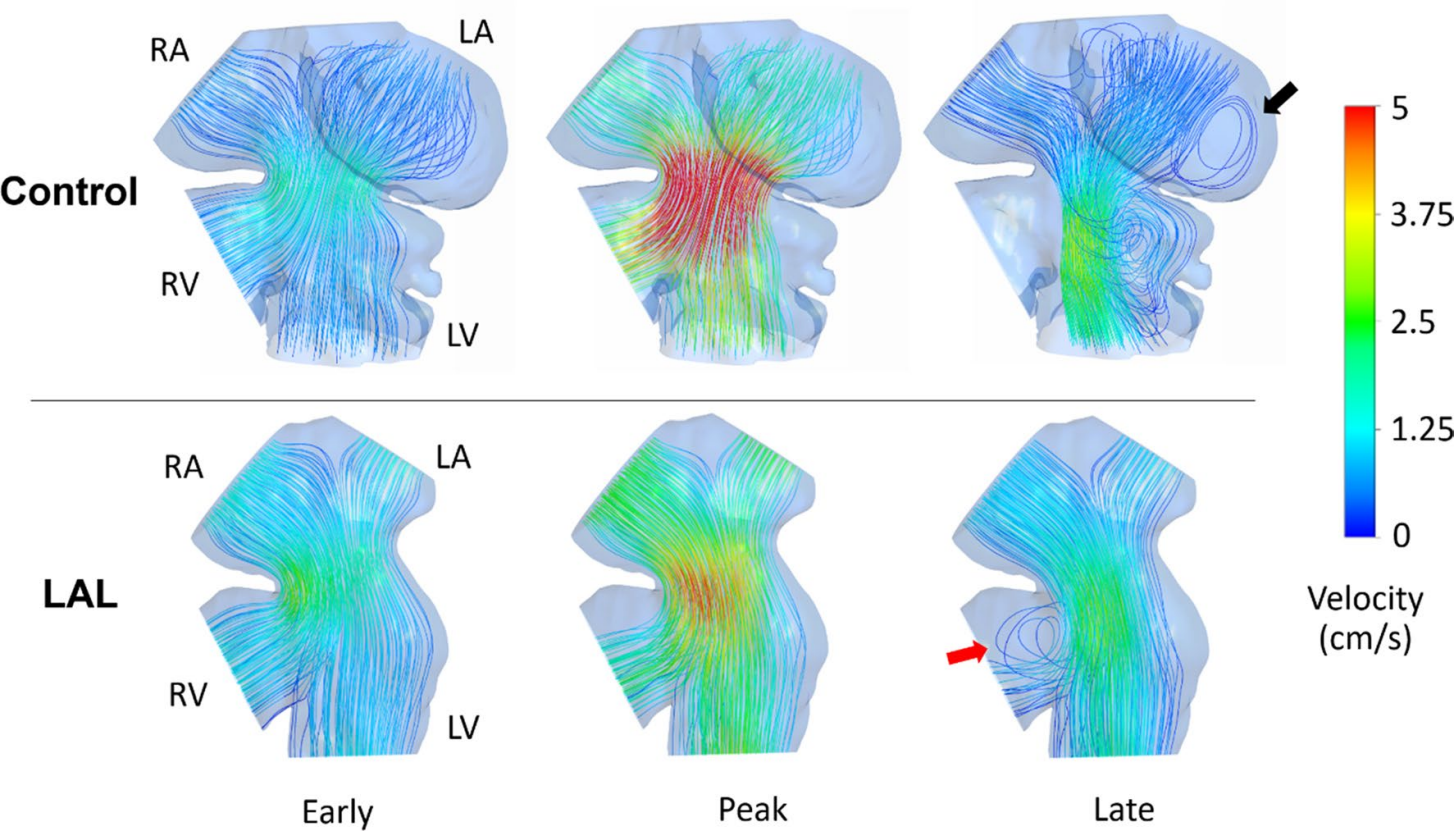
Velocity streamlines for HH21 LAL and control hearts considering different phases of cardiac cycle. Black arrow shows the recirculation in the LA of control heart which is not present in LAL heart. Red arrow shows the redirection of flow to RV after the LAL interference

Doppler velocity measurements (previously presented in Fig. 6) at HH21 reveal that the peak AV velocity is decreased after the LAL interference. However, time averaged velocities do not change in operated embryos, suggesting that the peak WSS in AV canal still reduces while the cardiac work is preserved. Reduced WSS levels are confirmed by the results of CFD simulations presented in Fig. 12. The velocity streamlines are also plotted for HH21 hearts as presented in Fig. 13. For the LAL heart, recirculating flow is not observed in LA and the flow is directed to RV at the diastolic phase of the cardiac cycle.

### HH30 LAL simulations

**Fig. 14:**
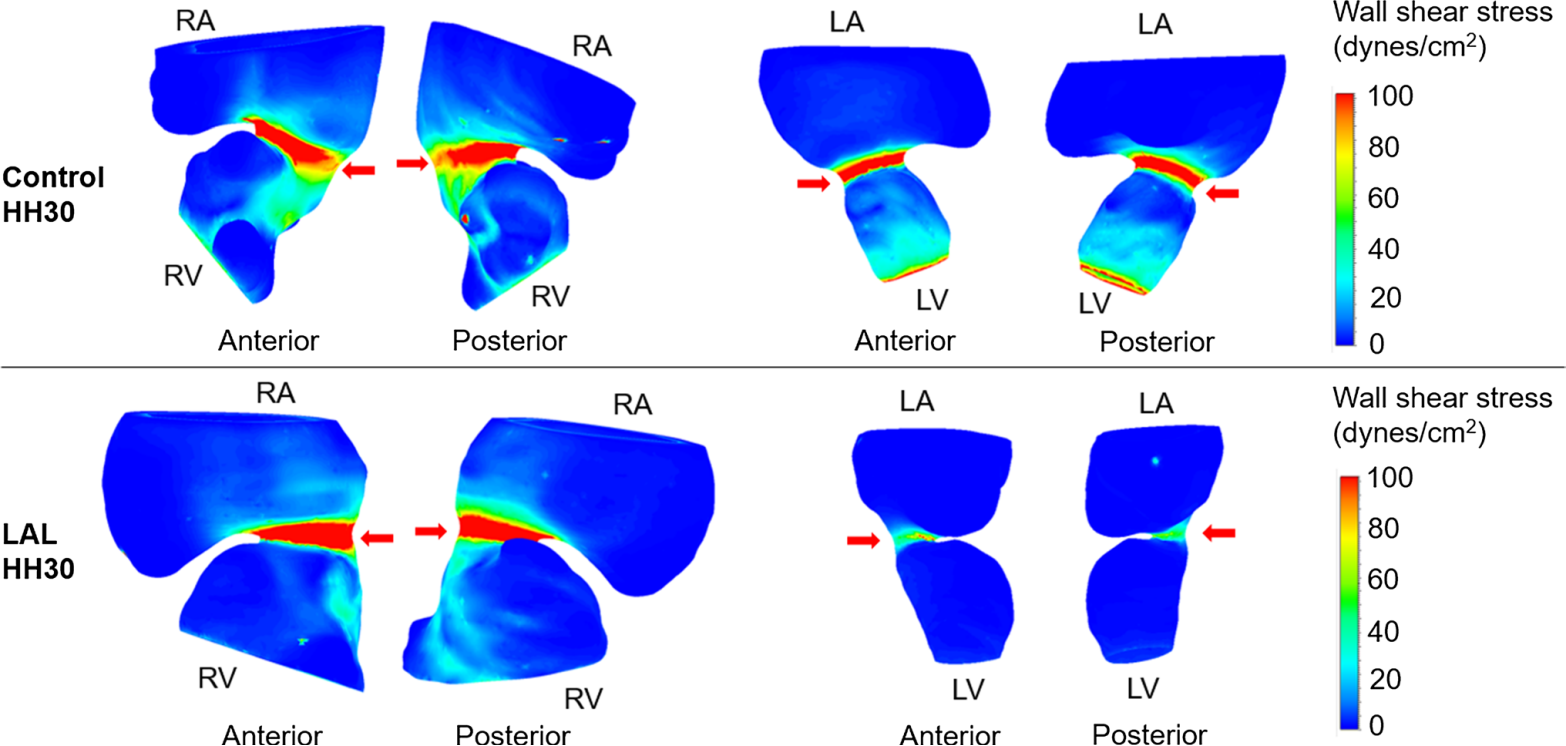
Peak WSS levels for septated HH30 hearts. Arrows show the AV cushions. After LAL interference, peak WSS on L-AV decreases while peak WSS on R-AV increases

As stated previously, separate models are generated for the left and right sides of HH30 hearts. LAL leads to a decrease in peak WSS on L-AV canal from 4.77 ± 0.59 Pa for controls to 2.13 ± 0.32 Pa for LALs (55.3% decrease). Average WSS on L-AV canal decreases from 0.76 ± 0.06 Pa for controls to 0.33 ± 0.013 Pa for LALs (56% decrease). On R-AV canal, LAL results in an increase in peak WSS from 5.3 ± 0.14 Pa for controls to 7.67 ± 0.39 Pa for LALs (44.7% increase). Average WSS on R-AV canal increases from 1.16 ± 0.047 Pa for controls to 1.44 ± 0.098 Pa for LALs (24.1% increase). For LAL embryos at HH30, WSS levels increase on R-AV canal, but significantly reduce on L-AV canal as presented in Fig. 14.

**Fig. 15:**
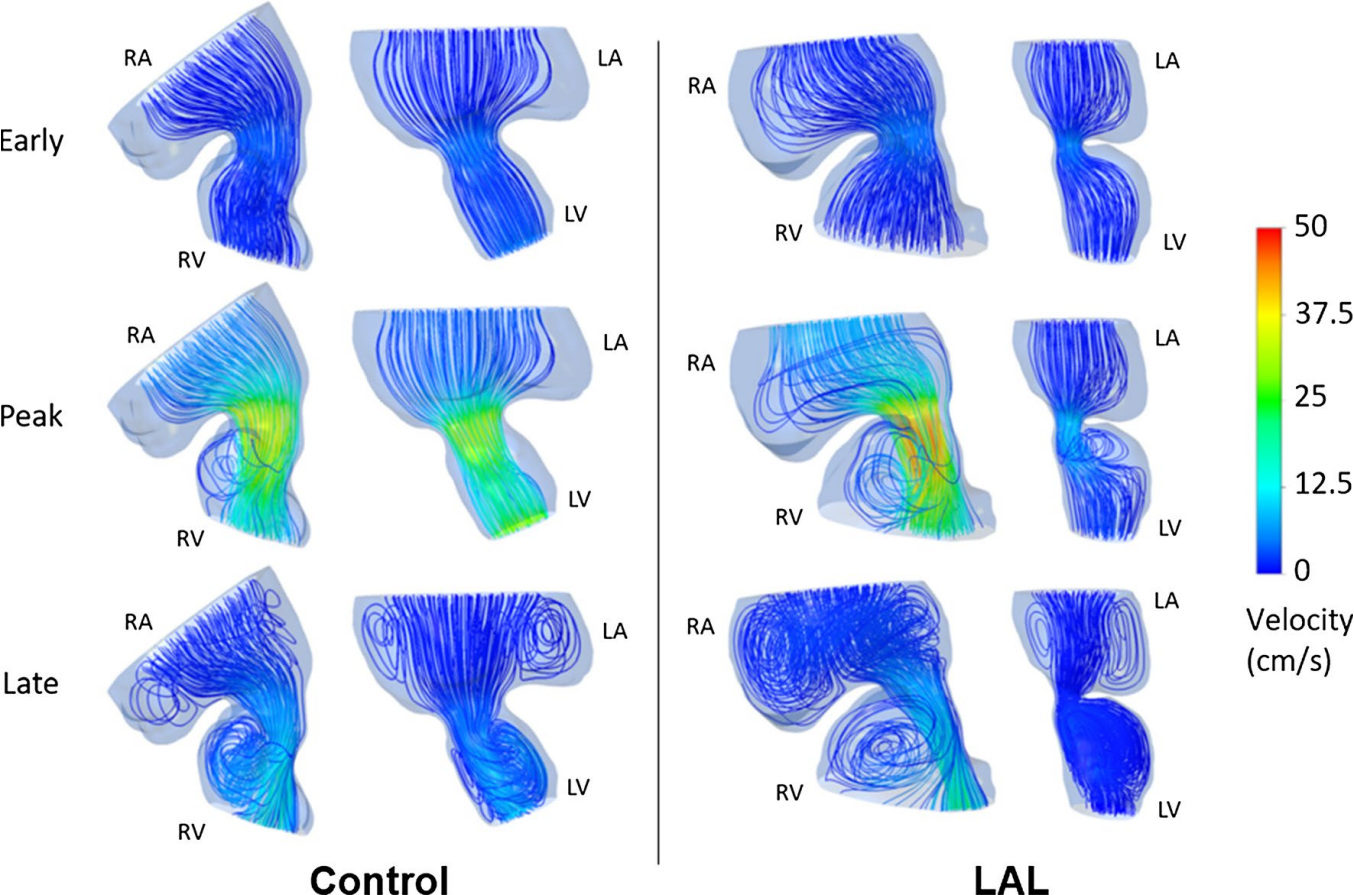
Velocity streamlines for HH30 LAL and control hearts for different phases of cardiac cycle. Flow velocities are significantly reduced in L-AV canal. LAL causes the flow to be redistributed to the right as well as the loss of circulatory flows in the late cardiac phase

Flow profiles are also examined at HH30 by separately modeling the left and right sides (see Fig. 15). The hemodynamic environment is found to be significantly different for LAL and control hearts, particularly at the peak systolic phase of the cardiac cycle. The findings show that LAL results in underdevelopment of left AV valve and overdevelopment of right AV valve at HH30.

**Fig. 16:**
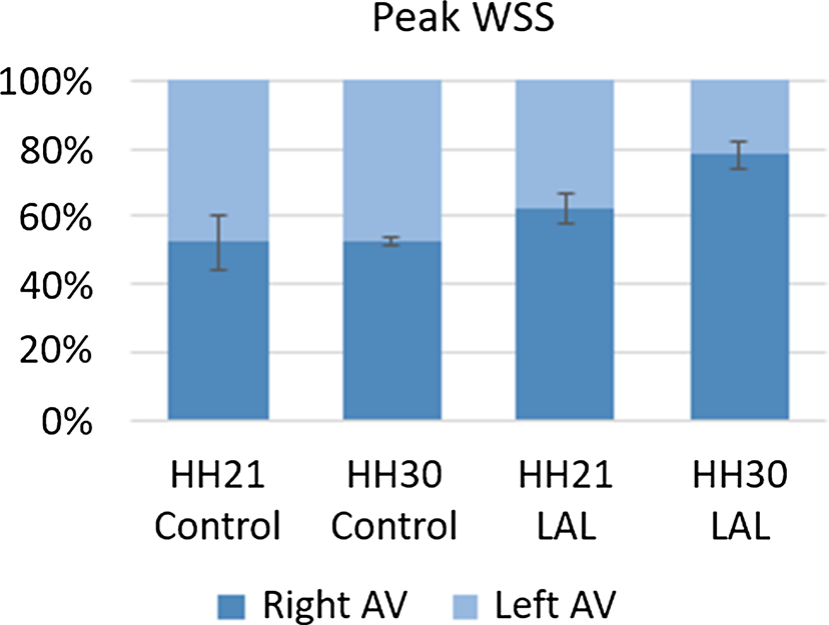
Balance of peak WSS between R-AV and L-AV cushions at HH21 and HH30

Peak WSS ratios between L-AV and R-AV cushions are compared for control and LAL embryos as shown in Fig. 16. The percentages given in Fig. 16 represent the left–right balance. The ratios are calculated by comparing the WSS levels on L-AV and R-AV cushions at a specific embryonic developmental phase; therefore the sum of percentages on the left and right side is always equal to 100%. The ratio of peak WSS is determined as 52.5 ± 8.15% to 47.5 ± 8.15% between R-AV and L-AV cushions of HH21 control hearts, respectively. This ratio is found as 52.6 ± 1.45% to 47.4 ± 1.45% between R-AV and L-AV cushions of HH30 control hearts, indicating that the WSS ratio is preserved during the normal embryonic heart development. LAL alters this WSS ratio at HH21, where the peak WSS ratio between R-AV and L-AV is determined as 62.5 ± 4.63% to 37.5 ± 4.63%, respectively. This fact shows that WSS on R-AV cushion is increased, and in the opposite way, WSS on L-AV cushion is reduced for HH21 LAL heart. WSS unbalance between AV cushions is even higher for HH30 LAL hearts, where the peak WSS ratio between R-AV and L-AV cushions of HH30 LAL heart is determined as 78.3 ± 4.01% to 21.7 ± 4.01%, respectively.

## Discussion

CHDs affect almost 1% of births per year. Previous studies have shown that perturbation of blood flow in the embryonic heart results in a spectrum of cardiac defects (Miller et al. 2003; Sedmera et al. 1999; Yalcin 2014), suggesting that disturbed hemodynamic environment is an important source for CHDs. HLHS, a serious type of CHD, accounts for approximately 25% of cardiac deaths in the first year of life (Bradley 1999). HLHS is a spectrum of cardiac malformations that are characterized by severe hypoplasia of the left heart-aorta complex resulting in progressive deterioration of LV function (Tchervenkov et al. 2000).

The LAL technique applied to the chick embryo is a surgical approach to examine the development of HLHS. LAL produces a phenotype that resembles HLHS including a severely underdeveloped LV. Partial ligation of LA reduces flow to the LV and redirects blood to the right side of the heart. Redistribution of hemodynamic load results in hypoplasia in the left side and hyperplasia in the right side of the heart (Hu et al. 2009; Midgett and Rugonyi 2014; Tobita and Keller 2000). The current study is designed to investigate disturbed hemodynamics in the developing heart following LAL performed at HH21 to provide insight into the progression of ventricular hypoplasia.

The disturbance in blood flow alters the biomechanical environment in the heart. Immediate effects are observed after the LAL surgery, including lack of circulatory flow in LA and redirection of flow to RV, particularly at the late diastolic phase. At HH30 stage, L-AV and R-AV canals are exposed to significantly different hemodynamic environments after LAL. The flow redirection to the right side leads to an increase in WSS on R-AV canal and a severe decrease on L-AV canal, which alters the shear stress balance in the heart.

LAL initiates an immediate WSS unbalance between R-AV and L-AV cushions, and this unbalance increases with the embryonic development. Histological assessments and micro-CT analysis reveal the underdevelopment of LV and L-AV valve for LAL embryos. It is considered that altered WSS environment plays a role in the progression of CHDs, and the early recovery of WSS unbalance between L-AV and R-AV cushions may slow down the progression of the initiated cardiac defects. Unbalance of peak WSS at HH21 is determined about 60-to-40 (right-to-left); however, this ratio reaches 80-to-20 (right-to-left) at the developmental stage of HH30. This fact indicates the need of repair to compensate the shear stress difference between the left and right sides of the heart. WSS levels are critically important for the heart development because of their important role in cell remodeling. WSS difference on the superior side between the control and LAL embryos is quite pronounced due to critically decreased WSS on LAL hearts. It is clearly understood that the reduced size of the superior AV cushions of LAL hearts has a direct relation with the lowered WSS levels on the superior side.

After LAL at HH21, stroke volume, cardiac output, ejection fraction, and time averaged velocity at AV canal do not change significantly; however, peak AV canal velocity decreases about 40%. When the flow velocities in HH30 LAL hearts are investigated, a severe decrease is observed in the L-AV canal. The velocities in R-AV canal do not show any significant change between the LAL and control embryos, showing that LAL interference does not impact the right heart development. On the other hand, the immediate drop of flow rate in the left side is still preserved at the embryonic stage of HH30, showing that the disruption in the left side cannot be recovered.

Similar results are also drawn by investigating AV canal orifice areas and cardiac outputs of HH30 control and LAL hearts. LAL causes a sharp decrease in L-AV canal cardiac output of approximately 47% and, in the opposite manner, an approximately 22% increase in R-AV canal cardiac output. The underdevelopment of L-AV canal leads to an excessive load on the right side of the heart which is promoting overdevelopment of R-AV canal. The increase in R-AV cardiac output is less severe than the loss in L-AV cardiac output. In need of a surgical operation, the surgery should be performed on the left side of the heart due to its less adaptive nature, which can be considered as a recovery operation to increase the amount of blood flow in LV.

Current therapy for HLHS is a three-stage reconstructive operation based on a single ventricle physiology, popularized by Norwood (Norwood et al. 1983). This life saving therapy has a relatively high mortality rate and can be offered in few centers. Alternatively, a prenatal surgical approach has been proposed to restore the defect in utero (Freud et al. 2014; Tworetzky et al. 2004), where fetal aortic valvuloplasty is applied to eliminate aortic stenosis. The aim from this surgical intervention is to restore ventricular hemodynamics by increasing volume load for the hypoplastic LV, which will potentially increase the ventricular growth and rescue hypoplastic left heart. In a clinical trial from Boston’s Children Hospital, partial restoration of the defect from prenatally operated patients has demonstrated that restoration of fetal hemodynamics for ventricular hypoplasia is a feasible approach to treat this condition in utero (Tworetzky et al. 2004). Prenatal surgical approaches on correction of hemodynamics to treat a cardiac defect in utero have better promise than postnatal approaches. This is because while the fetal myocardium adapts to augmented workload by cellular hyperplasia (i.e., increase in cell number), the adult heart adapts to this condition via cellular hypertrophy (i.e., increase in cell size) (Saiki et al. 1997; Sedmera et al. 1999). Therefore, restoring the hemodynamic loads in the heart of CHD patients before birth has a better chance of recovery compared to postnatal interference. For the development of novel prenatal surgical or therapeutic approaches based on restored hemodynamics, the first step is precise characterization of the hemodynamic environment under which the hypoplastic defects form and develop. Correlation of abnormal hemodynamic loads with altered biology causing the structural defects will contribute significantly to the understanding of how these conditions form in utero.

One major limitation in the study was the static geometries that were utilized in CFD models. It should be noted that the CFD results at the early and late cardiac phases include errors in terms of magnitudes of WSS and flow velocity, because a dynamic wall model is not employed to mimic the wall motions of the atrium and ventricle. The results presented at the peak systolic phase are more accurate compared to the results of early and late cardiac phases, because the static geometry used in the CFD model is the configuration at the peak systolic instant with fully dilated AV canal. Since the peak WSS levels are the main interest, the results of peak systolic phase are the focus of the investigation. A dynamic wall model is needed for better prediction of hemodynamics in the early and late cardiac phases to capture the effects of ventricle wall motion. Therefore, the static walls used in the current study lead to lower WSS and lower flow velocities at the early and late cardiac phases, due to the assumption of fully open valve in the models. On the other hand, the facts that we want to emphasize in the late cardiac cycle are the circulatory flows in the ventricle and the flow redirection to the right side of the heart. It is believed that both static and dynamic wall models will provide similar results in terms of the presence of the circulatory flows at the late systolic phase and the flow redirection to the right side of the embryonic heart. For a detailed investigation on the hemodynamics at the early and late cardiac phases, a CFD model with dynamic ventricle wall is planned to be employed in a future study.

In summary, the findings of the current investigation reveal the hemodynamics and structural alterations in the embryonic heart after LAL interference. Regarding the embryonic development of HLHS, correlations between disturbed hemodynamics and morphological growth caused by LAL are determined. The present study contributes to the field of cardiogenesis and highlights the critical importance of hemodynamics in the proper ventricular development.
